# Single versus dual innervation in facial palsy reanimation with free functional muscle transfers: a systematic review and meta-analysis

**DOI:** 10.1016/j.jpra.2025.06.009

**Published:** 2025-06-19

**Authors:** Andrin S Brader, Jonathan Leckenby, Adriaan O Grobbelaar, Cédric Zubler

**Affiliations:** a Department of Plastic and Hand Surgery, Inselspital University Hospital Bern, University of Bern, Freiburgstrasse 18, Bern, Switzerland; b Department of Surgery, Division of Plastic Surgery, University of Rochester Medical Center, 601 Elmwood Ave, Rochester, NY 14642, USA; c Department of Plastic and Reconstructive Surgery, Great Ormond Street Hospital for Children, Guilford St, London WC1N 3BH, United Kingdom; d Division of Surgery and Interventional Science, University College of London, Royal Free Hospital, 9th Floor (East), 2QG, 10 Pond St, London NW3 2PS, United Kingdom

**Keywords:** Facial palsy, Facial reanimation, Single innervation, Dual innervation, Bell’s palsy, Reconstructive surgery, Plastic surgery

## Abstract

**Background:**

Free functional muscle transfers (FFMTs) have emerged as the gold standard for reanimation of chronic facial palsy. However, the various options for neuronal input to power the transferred muscle remain an issue of debate, especially the question whether a single or two different nerves are used. The purpose of this study was to review the available clinical data on single versus dual innervation in FFMTs and compare their outcomes to better understand if dual innervation offers a significant benefit.

**Methods:**

A systematic review and meta-analysis were performed. Cochrane, EMBASE and PubMed MEDLINE database were searched following PRISMA guidelines 2020. All publications providing original clinical outcome data on dual nerve innervation of FFMTs in human patients were included.

**Results:**

The initial search yielded 451 studies of which 16 met the inclusion criteria for qualitative analysis and 4 for quantitative meta-analysis. A total of 98 patients were analysed, with 53 (51.94 %) women, mean age of 44.5 years in the single-innervation group and 41.2 years in the dual-innervation group, and an average follow-up period of 15 months. Both groups individually showed significant improvements (single innervation 41.1 %, *p* = 0.003, dual-innervation 50.7 %, *p* = 0.017). However, there is insufficient evidence to conclude a statistically significant difference in outcomes between the two methods.

**Conclusion:**

Both methods show meaningful improvements post-treatment. The dual innervation patients tended to have longer palsy durations and slightly better but more varied post-treatment improvements with overall no statistically significant difference to the single innervation group.

## Introduction

Facial palsy, a neuromuscular disorder resulting from impairment of the seventh cranial nerve can present with a variety of aesthetic and functional challenges. The dysfunctional neuronal input impairs various features of daily living such as facial expression, speech, emotional communication, and mastication. Therefore, patients suffering from facial paralysis experience significant psychological and physical morbidity with consequential impacts on their quality of life.^1^^,^^2^ Apart from congenital afflictions, various aetiologies can trigger the condition later in life, such as infections, tumours or trauma, yet most cases present as idiopathic (e.g., Bell’s palsy).^3^

The overall objective of treatment is the restoration of facial symmetry and dynamic function. Over the years, various approaches have emerged trying to restore the unique physiologic features of the facial nerve. Depending on the aetiology, spontaneous recovery is common, although often incomplete and associated with synkinesis and dyskinesis.^4^ Where spontaneous recovery is unlikely or has not occurred and non-surgical management has been exhausted, surgical management is often considered. In cases where prolonged denervation of the mimic musculature has resulted in irreversible muscle atrophy and loss of neuromuscular endplates, free functional muscle transfers (FFMTs) nowadays are regarded as the standard of care. Depending on patient characteristics and surgeon’s preference, the latissimus dorsi, gracilis, or pectoralis minor muscle is most commonly used for reanimation of the smile.^5^^,^^6^

Regardless of the muscle used for FFMT, one significant aspect of controversy is the concept of optimal neural input to power the transferred muscle tissue. There is an ongoing debate within the plastic surgery community regarding single versus dual innervation for the reanimation procedure.^7^^,^^8^ One can either use a single source, such as the masseteric or hypoglossal nerve or a cross-facial nerve graft (CFNG), for reinnervation or combine two nerve sources, typically a CFNG with an ipsilateral non-facial nerve.

Single innervation by an ipsilateral non-facial nerve allows for good voluntary control. It provides strong and fast muscle contraction.^9^^,^^10^ However, the goal is not only to allow for voluntary control, but to restore a spontaneous smile as a response to emotional triggers. In cases where a single non-facial nerve input is used, this requires significant brain plasticity and retraining. A CFNG from a contralateral branch of the facial nerve on the other hand allows for more spontaneity and synchronicity with the healthy side but only provides a much lower axonal count and therefore weaker neural input. Due to these limitations the concept of dual innervation was developed, with the idea of combining the benefits of different nerve sources. The idea is to use the neural input strength of an ipsilateral non-facial nerve and enhance spontaneity by adding a CFNG. However, there is a lack of scientific evidence regarding the mechanisms of dual innervation at the motor end-plate level.

Nevertheless, clinically dual innervation has assumed benefits (superior control over facial expressions, more natural appearance, faster recovery and more symmetrical facial movements further bearing reduced risk of synkinesis)^11^, ^12^, ^13^, ^14^, ^15^ but is also associated with increased surgical complexity. Some studies have described that dual innervation enables a more spontaneous smile through synchronization of the FFMT with the unaffected facial side, resulting in more natural and aesthetic pleasing results.^16^^,^^17^ Furthermore, this can also help bridge the time required for the axons to regrow through the cross-facial nerve and reinnervate the muscle.^18^

As both approaches show promising outcomes there is no consensus on either relative superiority.^19^

The ongoing debate surrounding both topics of efficacy and superiority in outcomes is in need for further research to ensure affected patients receive optimal care. Thus, the aim of our study was to critically evaluate the available evidence and compare post-operative results of single versus dual innervation in the treatment of facial palsy with FFMTs.

## Methods

A systematic literature review and meta-analysis was performed, following the PRISMA 2020-guidelines and the MOOSE-checklists.^20^ No formal ethics application was required for this study.

### Search strategy and selection criteria

Cochrane Library, EMBASE and MEDLINE (via PubMed) were searched on the 13th of March 2024. The following search strategy was used: ((facial palsy) OR (facial paralysis) OR (facial paresis) OR (facial nerve) OR (Bell* palsy)) AND ((Functional muscle transfer*) OR (free flap*) OR (muscle flap*) OR (free muscle*)) AND ((innervat*) OR (re-innervat*) OR (reinnervat*) OR (reanimat*) OR (re-animat*) OR (neural input*) OR (nerv*)) AND ((mixed) OR (double*) OR (triple) OR (cooptat*) OR (coapt*) OR (combin*) OR (simultaneous*) OR (two) OR (dual) OR (supplement*) OR (babysit*)). Only publications written in English and with available full text were considered in this review. There were no time period limitations for inclusion. After exclusion of duplicates, the remaining titles and abstracts located were screened (ASB, CZ). Papers of conflict were forwarded to a senior author for decision-making (AOG). Remaining studies were screened in full text by two independent reviewers (ASB, CZ). Only studies that provided original outcome data on single versus dual innervation of FFMTs in facial palsy patients were included. Any disagreements regarding the eligibility of full-text articles were again resolved by a senior author (AOG, JL). An extensive cross-check of the references from the original studies was performed to identify potential additional articles of interest. Studies not written in English, other reviews, case reports, animal studies, and cadaveric studies were excluded.

### Data extraction and statistical analysis

Two reviewers (ASB, CZ) independently performed the data extraction. The following baseline characteristics were chosen and extracted from the included studies: first author, year of publication, study design, prospective or retrospective, duration of study in months, multi- or monocenter study, number of patients, gender, mean age in years, mean preoperative paralysis time in months, number of patients per type of surgery (masseteric only, cross-facial nerve graft, cross-facial nerve graft with masseteric, free nerve transfer, spinal accessory nerve), reported outcome measurement, pre-operative score, post-operative score, mean postoperative follow-up time in months. Data collected from the included studies was then analysed for 3 different groups (overall data, single innervation group, dual innervation group) and reviewed by an external statistician. For each study, individual outcome measures were recalculated as proportional improvements from the reported preoperative baseline. Students’ *t*-tests and ANOVA-Tests in a general linear model were further used as applicable.

### Quality assessment

Two reviewers (ASB, CZ) assessed the methodological quality of the four included studies using the MINORS-criteria independently.^21^ Disagreements were resolved by consensus with AOG and JL.

## Results

### Literature search

**Figure 1:**
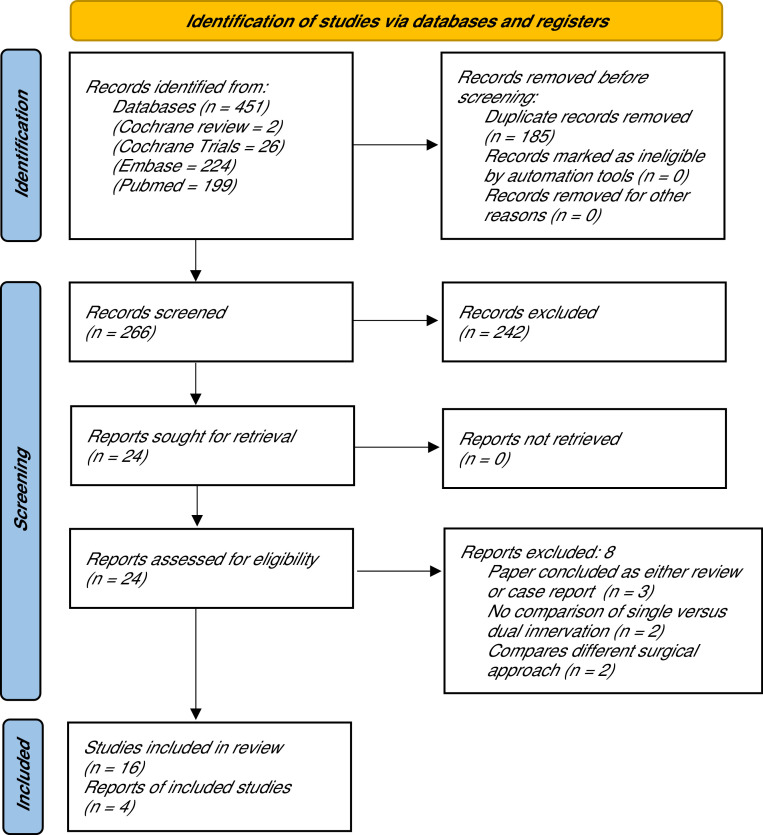
Flow diagram of eligible studies. After screening and applying exclusion criteria, 16 studies from the initial 451 items were included in this systematic review.

The initial search yielded 451 studies that were imported into Rayyan for further selection.^22^ 185 duplicate records were manually removed before primary review. The remaining 266 studies were subjected to a title and abstract screening by two independent reviewers (ASB, CZ) of which 242 were excluded. 24 papers underwent full text review. 16 papers provided original outcome data relevant for this review, while 4 studies qualified for quantitative comparison and meta-analysis. The search syntax is demonstrated in Figure 1.

### Characteristics of included studies

16 trials were included in this systematic review.^1^^,^^3^^,^^6^^,^^23^, ^24^, ^25^, ^26^, ^27^, ^28^, ^29^, ^30^, ^31^, ^32^, ^33^, ^34^, ^35^, ^36^ The characteristics of these studies are shown in Table 1. 4 studies were comparative studies, 12 were single cohort studies, all but one were retrospective studies and 5 were multi-centre studies. A total of 256 patients were included in these 16 studies, 71 in the single-innervation group (15 CFNG, 0 hypoglossal, 56 masseter) and 175 in the dual-innervation group (146 CFNG + masseter, 29 CFNG + hypoglossal). Patients were included into the studies over a mean period of 54.4 months. 93.75 % of studies reported postoperative follow-up time, which was 15 months on average with a range from 6 to 36 months.

**Table 1:** Characteristics of the 16 included studies.

	Type of study	Design	Location	No. Patients	Men (%)	Female(%)	Mean age (years)	Mean follow up time (months)	Technique used	Reported outcome measure
Sakuma^1^	Comparative	Retrospective	Monocentric	18	7 (38.89)	11 (61.11)	34.2	74	1c; 2a	Spontaneous smile grading scale
Cardenas-Meja^25^	Cohort	Retrospective	Multicentric	9	6 (66.67)	3 (33.33)	37.6	36.7	2a	Terzis Score
Biglioli^26^	Cohort	Retrospective	Multicentric	4	1 (25)	3 (75)	49.4	18	2a	House-Brackmann Score
Sforza^27^	Cohort	Retrospective	Multicentric	13	3 (23.08)	10 (76.92)	41	17	2a	House-Brackmann score
Watanabe^28^	Cohort	Retrospective	Monocentric	3	2 (66.67)	1 (33.33)	47	NR	2a	Harii score
Thachil^24^	Comparative	Retrospective	Monocentric	47	26 (55.32)	21 (44.68)	58 (single); 31.5 (dual)	26.83 (single); 26.52 (dual)	1c; 2a	Photograph analysis
Wen^30^	Comparative	Retrospective	Monocentric	14	3 (21.43)	11 (78.57)	63 (1-stage); 27 (2-stage)	26.5 (1-stage); 26.6 (2-stage)	2a	Photograph analysis
Okazaki^32^	Cohort	Retrospective	Monocentric	4	2 (50)	2 (50)	44	24	2a	Harii score
Park^6^	Comparative	Retrospective	Monocentric	21	10 (47.62)	11 (52.38)	41.4	12	1c; 2a	Terzis score and photograph analysis
Tzafetta^31^	Cohort	Retrospective	Monocentric	11	5 (45.45)	6 (54.55)	32	36	2a	House-Brackmann Score
Watanabe^29^	Cohort	Retrospective	Monocentric	7	1 (14.29)	6 (85.71)	50.7	27	2a	Harii/House-Brackmann score
Dusseldorp^23^	Cohort	Retrospective	Monocentric	25	12 (0.48)	13 (0.52	38.4	9.6	1a; 1c; 2a	eFACE
Uehara^34^	Cohort	Retrospective	Monocentric	6	3 (50)	3 (50)	56.7	18	2a	Photograph analysis
Boahene^3^	Cohort	Prospective	Multicentric	12	2 (16.67)	10 (83.33)	46	12	1a; 1c; 2a	Photograph analysis
Amer^35^	Cohort	Retrospective	Monocentric	29	18 (62.1)	11 (37.9)	22.76	24	2a	SMILE system
McNeely^36^	Cohort	Retrospective	Monocentric	9	4 (44.44)	5 (55.56)	8.6	27.3	1a; 1c; 2a	House-Brackmann Score

1a, single innervation–CFNG; 1b, single innervation-hypoglossal; 1c, single innervation–masseteric; 2a, dual innervation–CFNG+Masseteric; 2b, dual innervation; CFNG+Hypoglossal.

### Quantitative analysis

Given the heterogeneity of studies, surgical techniques and outcome-measurements used, studies were further selected for inclusion in quantitative evaluation and meta-analysis. The primary goal was to compare single versus dual innervation outcomes in FFMT for facial palsy reported in the literature. To improve comparability, only studies that provided original outcome data on both single as well as dual innervation patients were considered. This left us with cohorts of patients in which the same surgeons performed both techniques, effectively minimising potential differences due to surgical expertise or postoperative rehabilitation protocols. Furthermore, to eliminate bias due to varying neural input, only the two most frequently used techniques were compared. This left us with four studies^1^^,^^3^^,^^6^^,^^25^ that reported results from masseteric nerve alone (single innervation) as well as masseteric nerve combined with a CFNG from the contralateral facial nerve (dual innervation). By focusing on studies that meet these criteria, confounding factors were minimised. Thus, isolating the impact of single versus dual innervation on functional outcomes. The different scales used for reporting outcomes in the included studies necessitated a standardisation process. To ensure comparability of the different outcome measurements, they were converted into percentages of improvement relative to the reported preoperative baseline data. This enabled the assessment of relative functional changes, mostly independent of specific scales or metrics used and allowed for direct comparison between studies.

The assessment of the methodological quality of these four included studies is illustrated in Table 2.

**Table 2:** Methodological quality of included studies^1^^,^^3^^,^^6^^,^^25^ using the methodological index for non-randomized Studies (MINORS).^21^

Criterion	Boahene et al.	Park et al.	Sakuma et al.	Thachil et al.
A clearly stated aim	2	2	2	2
Inclusion of consecutive patients	1	1	1	1
Prospective collection of data	1	1	1	1
Endpoints appropriate to the aim of the study	2	2	2	2
Unbiased assessment of the study endpoint	1	1	1	1
Follow-up period appropriate to the aim of the study	2	1	2	2
Loss to follow-up <5 %	1	1	1	2
Prospective calculation of the study size	0	0	0	0
Power analysis				
Adequate control group	2	2	2	2
Contemporary study groups	2	2	2	2
Baseline equivalent of groups	2	2	2	2
Adequate statistical analysis	1	1	1	2
**Total score**	**17**	**16**	**17**	**19**

Bolded values relate to ‘Total score’.

Table 3 provides a descriptive overview of the single and dual innervation cohorts. The number of patients in the single innervation group varied quite significantly between studies, with an average number of 14 patients. In contrast, the dual innervation groups were smaller, with an average of 10.5 patients, but demonstrated greater consistency in patient numbers.

**Table 3:** Descriptive statistics for patients classified in either one of the categories: single- or dual-innervation across the four qualifying studies.

	Minimum	Maximum	Mean	Std. deviation
Total patients Single Innervation	7	33	14.00	12.675
Male single innervation	1	18	6.50	7.767
Female single innervation	4	15	7.50	5.066
mean age single innervation	34.2	58.0	44.467	12.2317
Mean duration of palsy (months) - Single	11.8	97.2	67.333	48.1391
Improvement post vs pre (%) - single innervation	28.705	50.000	41.09575	9.017465
Total patients dual innervation	5	14	10.50	4.041
Male dual innervation	1	6	3.50	2.082
Female dual innervation	4	11	7.00	2.944
Mean age dual innervation	31.5	50.5	41.167	9.5044
Mean duration of palsy (months) - dual	70.8	138.0	108.600	34.3785
Improvement post vs pre (%) - dual Innervation	25.380	76.000	50.74800	21.096295

The gender distribution indicates that the mean number of males in the single innervation group (6.5) was higher than in the dual group (3.5). The mean age of patients was overall comparable, with the single innervation group averaging 44.5 years and the dual innervation group at 41.2 years.

However, there was a notable discrepancy in the duration of palsy between the two groups. The mean duration of palsy in the single innervation group was 67.3 months (equivalent to around 5.5 years), while the dual innervation group exhibited a substantially longer duration, with an average of 108.6 months (approximately 9 years). This suggests that cases of dual innervation often involved longer-standing palsy. Whether the patients suffered from complete or incomplete facial palsy, and especially the distribution of the remaining function was not uniformly reported in the included studies.

One-sample *t*-tests were used in Table 4 to ascertain whether the mean improvement percentage in post-treatment scores for the single and dual innervation groups is significantly different from zero. The analysis yielded a t-value of 9.115 for the single innervation group with a p-value of 0.003, indicating a highly significant result. The mean improvement was 41.09 % (95 %CI: 26.74 % to 55.44 %). Similarly, the *t*-test yielded a t-value of 4.811 and a p-value of 0.017 for the dual innervation group, indicating a statistically significant improvement. The mean improvement in this group was higher, at 50.74 % (95 %CI: 17.18 % to 84.31 %). The wider confidence interval indicates a greater degree of variability in treatment responses, yet the observed improvement remains statistically significant. This demonstrates the effectiveness of the individual interventions for both the single and dual innervation group.

**Table 4:** One-sample *t*-test for analysis of mean overall improvement percentages in outcome measurement scores for both groups.

*One-sample test*						
	Test value = 0					
	t	df	Sig. (2-tailed)	Mean difference	95% confidence interval of the difference	
					Lower	Upper
Improvement post vs pre (%) - single innervation	9.115	3	.003	41.09	26.74	55.44
Improvement post vs pre (%) - dual innervation	4.811	3	.017	50.74	17.18	84.31

**Figure 2:**
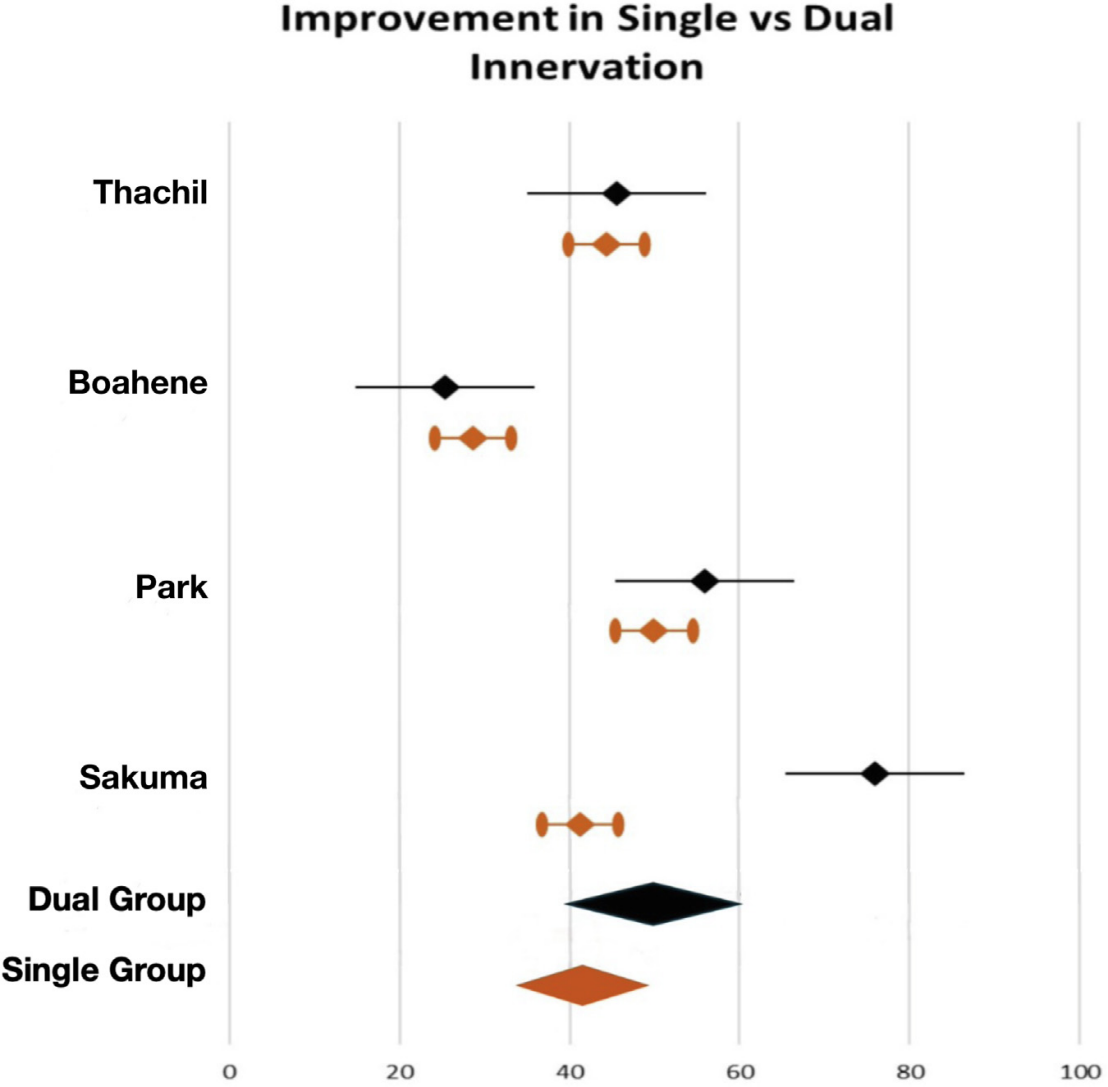
Visualisation of the reported postoperative improvement of included studies in percentage. Single innervation results are displayed in brown, dual innervation in black.

Figure 2 compares the perioperative improvements for both treatment options of the included studies as well as for the pooled single versus dual innervation groups. For patients in the single innervation group an average improvement of 41.1 % was reported, with consistent results across cases, whereas the patients in the dual innervation group demonstrated a higher mean improvement of 50.7 %, albeit with greater variability and no statistically significant difference.

Overall, the dual innervation group tended to have longer palsy durations and slightly better, albeit more varied, post-treatment improvements than the single innervation group.

## Discussion

The goal of facial reanimation with FFMT is to restore static and dynamic facial symmetry and to allow patients to regain a spontaneous smile, optimally with the lowest burden in scars, donor site morbidity and surgical time needed. Whether single or dual innervation is superior for this task remains a matter of debate. Both techniques show remarkable outcomes but are afflicted with specific limitations and drawbacks.

Single innervation techniques rely on one robust non-facial nerve as source for reinnervation of the free muscle transplant such as the hypoglossal or masseteric nerve. Advantages lie in reliability, simplicity, and the amount of muscle contraction the nerve can provide. The short distance of neural regrowth and the high axonal count results in fast reinnervation and good strength of the transferred muscle. Due to this, the masseteric input is often the method of choice in older patients. However, one must often accept limitations such as a lack of spontaneity in facial movements and less emotion-driven smiles. This may further impact the emotional and psychosocial expression.^23^

Cross-facial nerve grafts take time for axonal regrowth and provide a lower axonal count. Due to the growth across the nerve graft, delays of reinnervation and therefore spontaneous movement can appear, relative to the length of the nerve graft, which is why these are usually performed in stages.^6^ However, it may provide a more spontaneous and natural smile and is therefore used especially in younger patients as a single-innervation input.

The dual-innervation techniques have evolved as an effort of harvesting the advantages of both regional as well as CFNGs, providing patients with fast and strong reinnervation, yet also spontaneity. One question remaining is the impact of timing of these axons reaching the neuromuscular endplates. Especially, one may doubt the impact a CFNG will have if its axons reach the neuromuscular endplates after a much higher count-input such as the masseteric nerve.

Some cohort studies have been published that claim both functional and aesthetic superiority of dual-innervation results. Dusseldorp et al.^23^ demonstrated more oral commissure movement during spontaneous smiling compared to single-innervated patients. Similarly, Sakuma^1^ and Thachil^25^ found that patients that received dual-innervation had significant improvements in teeth exposure during spontaneous smiling and better commissure height compared to single-innervation.

Yet the evidence remains thin. Our meta-analysis was unable to demonstrate any statistically significant differences in overall outcome between single and dual innervation FFMT. While the mean improvement in dual innervation patients was slightly higher, this group also displayed a higher variability in outcome. Apart from surgical technique, this may also indicate greater diversity in the baseline severity or differences in the underlying pathology, operator experience, or intrinsic mechanisms of nerve regeneration. This underscores the fundamental gaps in our understanding of the biological processes underlying functional recovery, including reinnervation dynamics and synaptic plasticity in nerve regeneration. Experimental studies linking neurobiological mechanisms with clinical observations are essential and will help us optimise surgical approaches. This is especially relevant because apart from the assumed benefits, dual innervation also has some downsides. The technique may bear greater risks, as it is more complex, takes more time and requires additional scars and donor sites compared to a single ipsilateral non-facial input.

Assessing the post-surgical outcome in facial palsy reanimation is a complex task and multiple grading systems are currently in use. In clinical practice, the House-Brackmann and the Sunnybrook-facial grading System are the most frequently used. With the House-Brackmann scale, the advantages lie in a good overall evaluation of function and degree of paralysis. Yet it lacks in differentiation of more dynamic or subtle changes. It is therefore not optimal in assessing detailed outcome changes following surgery.^37^

Often favoured for its sensitivity, applicability and easy use, the Sunnybrook-Facial-Grading-System has been developed. It offers a more precise measurement of change for resting symmetry, synkinesis and voluntary movement.^38^ Especially in cases involving synkinesis or in the paediatric populations this grading system is therefore often favoured.

Another widely used assessment tool is the Terzis-Grading-scale. Allowing specifically evaluation of outcomes after surgical reanimation procedures. However, since more data and more time are needed for evaluation, it is not widely used in routine clinical practice.^39^

Since time efficiency and inter-rater reliability are important considerations, automated analysis systems (e.g. eFACE) and other computerized tools (Facogram) offer potential advantages in reducing subjectivity and clinician time.^40^ Furthermore, such standardised systems could allow for greater comparison of data.

However, none of these systems is currently universally accepted and each one bears limitations. Additionally, due to small sample sizes in several studies, heterogeneity within patient populations and different follow-up durations, limitations occur even when comparing results assessed using the same grading system. Nevertheless, implementation of a universally accepted tool for outcome measurement would greatly advance the discussion and improve our understanding regarding the optimal treatment options for these patients.^40^

Nowadays, the choice between dual- and single-innervation methods is mostly influenced by patient factors and surgeon’s preference. Especially in younger patients who are under greater social pressure with regards to interaction and facial expression, and which exhibit better potential for nerve regeneration, dual innervation may be the preferred option. In contrast, older patients may benefit from a simpler, single innervation procedure. However, despite the increasing clinical relevance also in the paediatric population, there is a paucity of research investigating its long-term effects and either dominance of nerve input in this population. Moreover, there is an absence of literature directly comparing one-stage surgery using a long nerve to the contralateral facial nerve (Harri/Takajashu-technique)^41^ with dual innervation.

### Limitations

Due to the nature of the available literature, this study is encumbered by several limitations. Firstly, the overall number of studies directly comparing both techniques remains limited, particularly in specific subgroups (e.g., paediatric patients). Furthermore, study design, patient populations, surgical techniques, institutional protocols, and follow-up durations vary greatly between studies. Differences in nerve inputs and type of muscle used complicates direct comparisons and limits the generalisability of the findings. This is why we decided to limit quantitative analysis to the most commonly reported techniques, masseteric nerve alone versus masseteric nerve combined with a CFNG. Furthermore, most published studies are retrospective, which introduces potential bias.

Another important limitation is the lack of standardised outcome measures and post-operative scoring system across studies. While some studies assess facial reanimation outcomes using newer objective tools such as Emotrics or 3-D motion analysis, others rely on subjective grading scales like the Sunnybrook or House-Brackmann systems. This heterogeneity practically makes any direct comparison impossible and necessitated a standardisation process. Using the pre- to postoperative improvements allowed such a quantitative comparison, while only including studies that reported both on single and dual innervation patients, reduced differences in reporting standards between the two groups.

Future research should focus on high-quality, prospective comparative studies with standardized outcome measures and long-term follow-up to better define the role of single and dual innervation in facial reanimation. Developments of facial reanimation may lie in hybrid innervation techniques. With new nerve coaptation methods, newer technologies such as robotic assisted surgery or improved surgical planning with 3D modelling, artificial intelligence or virtual reality, surgical outcomes could potentially be further improved. Yet, we first need to improve our understanding of the interplay of axons of different nerves at the neuromuscular junction during regeneration, as well as overcome self-imposed limitations such as inhomogeneous outcome reporting.

## Conclusion

No significant differences between single and dual innervation in FFMT for facial palsy reanimation surgery were found with regards to the reported postoperative improvement. Both methods allow for significant improvements in patients with long-standing facial palsy. Further, both exhibit potential limitations and drawbacks. Choosing the best option for the patient may depend on different factors such as the age of the patient, the underlying cause of the palsy and specific surgical factors. The existing literature as shown in this study is inconsistent and definitive deductions are hindered by varying outcome measures.

## Author contributions

Conceptualization, AOG, JL and CZ; methodology, JL, AOG, ASB and CZ; formal analysis, ASB and CZ; investigation, ASB and CZ; data curation, ASB and CZ; Writing - original draft preparation, ASB and CZ; writing - review and editing, AOG and JL; visualization, ASB; supervision, AOG and JL; project administration, ASB. and CZ. All authors have read and agreed to the published version of the manuscript.

## Funding

The authors have not received any financial support for neither the research, authorship, nor publication of this article.

## Declaration of AI and AI-assisted technologies in the writing process

No AI-assisted technologies were used in the writing process.

## Informed consent statement

Not applicable.

## Ethical approval

Due to the nature of this study, no formal approval from an institutional review board or ethics committee was needed.

## Informed consent

No informed consent was in the study, including for the publication of identifying information or images.

## Authorship statement

The authors confirm that all co-authors have made significant contributions to the study and have approved the final manuscript.

## Declaration of competing interest

The authors declared no potential conflicts of interest also with respect to their authorship, search, and publication of this article.
